# Design and Realization of an Electromagnetic Guiding System for Blind Running Athletes

**DOI:** 10.3390/s150716466

**Published:** 2015-07-08

**Authors:** Marco Pieralisi, Valerio Petrini, Valentina Di Mattia, Giovanni Manfredi, Alfredo De Leo, Lorenzo Scalise, Paola Russo, Graziano Cerri

**Affiliations:** 1 Department of Information Engineering, Università Politecnica delle Marche, via Brecce Bianche, Ancona 60131, Italy; E-Mails: marco.pieralisi@univpm.it (M.P.); v.petrini@univpm.it (V.P.); g.manfredi@univpm.it (G.M.); a.deleo@univpm.it (A.D.L.); paola.russo@univpm.it (P.R.); g.cerri@univpm.it (G.C.); 2 Department of Industrial Engineering and Mathematical Science, Università Politecnica delle Marche, via Brecce Bianche, Ancona 60131, Italy; E-Mail: l.scalise@univpm.it

**Keywords:** electromagnetic sensors, aid for visually impaired athletes, antenna design

## Abstract

Nowadays the technologies aimed at improving the quality of life of people affected by visual diseases are quite common; e.g., devices to support walking or reading. Surprisingly, there is a lack of innovative technologies aimed at helping visually impaired athletes during physical activities. An example is represented by blind runners who need to be physically linked to a sighted guide by means of non-stretchable tethers during races; with consequent limitations in terms of performance and independence. This paper wants to investigate the possibility of realizing a system able to guide blind runners along a complex path, paving the way for the realization of an innovative device designed to improve their independence during training or competitions. The system consists of: (1) a mobile unit, which is placed before the runner and generates two “electromagnetic walls” delimiting the way; (2) a receiving unit (worn by the athlete) that provides vibro-tactile warnings every time the user is going outside the safe area so as to encourage him to move toward the central position. The feasibility and the utility of the system proposed are demonstrated by means of tests carried out thanks to the collaboration of a blind volunteer.

## 1. Introduction

In the world, 285 million people are estimated to be visually impaired: 39 million are blind and 246 have low vision [1]. Nevertheless the most common and largely used device is still the white cane because it is easy to use, cheap, and widely accepted among blind community, many technologies have been proposed, both in literature and on the market, to improve the autonomous mobility of people affected by visual diseases. They are mainly based on ultrasonic or optic sensors [2,3]. Anyway, whatever physical quantity they are based on, they present many limitations and no one of them meets either the international guidelines defined for ETAs [4], or the requests of visually impaired users. For this reason, new techniques are under investigation, as those based on electromagnetic fields, whose advantages with respect to the traditional ultrasonic and optic devices are widely explained in [5]. 

Surprisingly, there is a lack of innovative technologies aimed at helping visually impaired athletes during physical activities, even it is commonly known that sport is an ideal means to promote the integration of disabled people in general and blind people in particular. Sport can help people overcome their disabilities by strengthening their self-esteem and their ability to face difficulties. It is equally true that sight plays an extremely important role in almost every sport. For this reason, the most popular sports among blind people are those specifically thought for people affected by visual diseases (e.g., Torball and Goalball).

Nevertheless, the number of visually impaired people playing many other sports is increasingly growing. Some examples are athletics, judo, swimming, skiing, and football. A visually impaired athlete can play many of them on his/her own, using suitable supports or following the vocal direction of a sighted guide. For example there are no particular difficulties for swimming where, thanks to floating lanes, the races can be played with all the athletes together. A unique special precaution is to allow the coach to touch the athlete on the head with a small stick to indicate that the end of the pool is approaching and to allow the preparation for the turn. As concerns skiing, the modern technique used is based on a radio-guided communication: the instructor gives orders to the blind via a transceiver connected to a radio-headset placed on the head of the athlete. 

In some sports, such as running, visually impaired athletes still need to be physically linked to a sighted guide by means of a non-stretchable tether tied around their wrists or held between their fingers so as the distance between them does not exceed 50 cm [6]. Although many guides are provided with an excellent preparation, the blind athlete’s performance could be limited by the presence of the sighted guide. This is mostly true for long races, such as marathons, during which blind athletes need to change more than one guide with evident disadvantages and performance limitations.

In literature, few examples of technologies aimed at improving independence of blind athletes can be found. For example, Balmer proposed a system based on the measurement of the magnetic field produced by a wire properly laid along the side of the track [7]. The field measured is then processed and compared with a reference signal in order to obtain information about athlete’s position relative to the correct track and to produce a suitable signal to alert the runner about the correct direction to follow. The main disadvantage of such a system is the use of acoustic warnings. In fact, it is commonly known that vibration warnings are to be preferred since auditory feedbacks occupy user’s acoustic channel with the possibility of confusing and annoying him. Another recently developed device is the Remote Guide [8] able to guide the blind person in the correct direction through auditory and vibrational sensors used to communicate the directions given remotely by a sighted guide. It is clear how this device is able to avoid the presence of the guide linked to the athlete during race, but it does not improve the independence of blind runners in a significant way and still presents limitations due to the necessity of following the vocal directions of a coach.

Another interesting work is the one proposed in 2005 by Hosseini *et al.*, concerning the realization of an electrical system to make blind people capable of safely navigating and quickly avoiding obstacles and other hazards while riding a bicycle without any other help [9]. According to the authors, an interesting application of this system could be in Paralympic sports as a new method for quadriplegics and blind people. 

In this context, the main aim of this paper is to investigate the possibility of designing a system able to help a visually impaired person walking or running autonomously, by guiding him/her along a desired path. In particular, the system described in the following is based on electromagnetic (EM) technology. Obviously the introduction of a new technology during official races would require a complete review of the rules of the game. Therefore, as first step, it could be interesting to propose the system as a support for training, which also needs the presence of a sighted guide. 

The motivation behind the choice of designing a system based on EM fields lies in a previous study conducted by the same authors to demonstrate the feasibility of using EM technology to realize a travel aid to support the autonomous mobility of visually impaired and blind people [10]. In order to actually investigate the performances of such a system, many tests have been carried out with the collaboration of a blind marathon athlete [11]. Moved by positive results and feedback, we decided to develop an EM system to support him during his daily running, improving his independence during the training.

**Figure 1:**
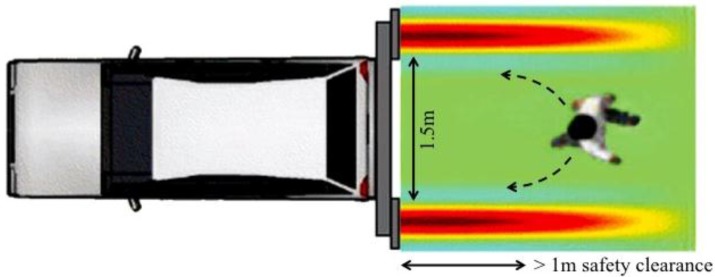
Schematic representation of the guiding system: two transmitting antennas, set on a mobile unit, generate two EM walls delimiting a virtual hallway that can guide the running athlete, properly equipped with the receiving system, along whatever paths.

Therefore, as Figure 1 shows, the idea has been to equip a vehicle (that is depicted as a car, but it might be a bike or generally a mobile unit) with two transmitting elements able to generate two radiation patterns shaped as narrow vertical fans (angular width < 10°) delimiting two invisible ‘EM walls’ that confine the runner inside a secure hallway. In order to make sure that the athlete always remains inside the virtual hallway, two vibro-tactile warnings are generated each time he is getting close to one of the borderlines so as to encourage him to move back toward the central position, where no more warnings are produced. In a few words, as the mobile unit goes forward, the runner may safely follow it along a desired path.

It is worth noting that to use the guiding system along complex paths, characterized by tight curves and sudden directional changes, the radiation of wide vertical fans (angular width of about 30° over horizontal plane, typical of commercially available horn antennas) should be preferred. In fact if the EM walls are too narrow, it could be happen that during a sudden tight curve the athlete crosses the EM wall in such a short time that the receiving unit does not have the time to warn him. On the contrary, bearing in mind the desired application of such a technology, that is along athletic tracks (that means no tight curves), the choice of generating narrow EM walls should be preferred because of the possibility to confine the EM fields inside small and well defined regions. To this end two antenna models have been optimized to create *ad hoc* transmitting and receiving sensors. Moreover, the whole system has been set up using laboratory instrumentation and finally tested thanks to the collaboration of a blind marathon athlete.

It is important to underline that the aim of this work is not introducing any novelty in antennas and circuits design, but to use the existing EM theory and technologies to propose an innovative application able to improve the quality of life of athletes affected by visual diseases. 

The paper is organized as follows: in Section 2, the whole system proposed is explained in detail, focusing the attention on the two subunits and in particular on the antennas design and realization. In Section 3, the tests carried out with the blind volunteer are described. In Section 4, some important suggestions for future developments are discussed. Finally, Section 5 summarizes all the results obtained so far.

## 2. Experimental Section

### 2.1. System Description

**Figure 2:**
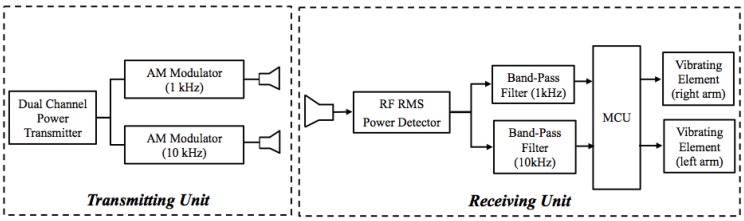
Schematic representation of the whole system, divided in two subunits: one transmitting unit moving in front of the user, and one receiving unit worn by the user and including two vibrational warnings.

The EM guiding system consists of a transmitting unit to be placed on a mobile structure running in front of the user and a receiving sensor worn by the athlete. Figure 2 depicts a schematic representation of the whole system: the transmitting unit, on the left, includes a X-band signal generator, two radiating elements that generate the two EM walls and two modulators at two different frequencies in order to discriminate left and right boundaries. The choice of the transmitting frequency is essentially related to the possibility of designing antenna with reduced dimensions, whereas the choice of the modulation technique is not critical. In particular, a square wave amplitude modulation (AM), duty cycle 50%, at the frequencies of 1 kHz and 10 kHz, for the left and right EM walls respectively, have been used. The receiving system, on the right of Figure 2, consists of a small antenna, a demodulator, two band-pass filters, a Micro Controller Unit for thresholds setting, and further signal processing and two vibration transducers to communicate a warning signal to the blind athlete. Among all the components, the antennas represent the most important elements whose design needs to meet specific requirements. In the following, both transmitting and receiving units will be described in detail, paying special attention to the antennas design and realization.

#### 2.1.1. Transmitting Unit

The transmitting subsystem is responsible for signal generation and radiation. The main elements of the transmitting unit are the two antennas (one for the left boundary and the other for the right one) that generate the two lateral EM walls by radiating two vertical fan beams: narrow over the horizontal plane and wide over the vertical plane. In detail, the main requirements for the transmitting antennas are referred to the horizontal plane (*i.e.*, H-plane): a Half Power Angular Beam Width (HPBW) in the range 5° < HPBW < 10° and a Side Lobe Level (S_LL_) < −20 dB so as to avoid undesired warnings.

**Figure 3:**
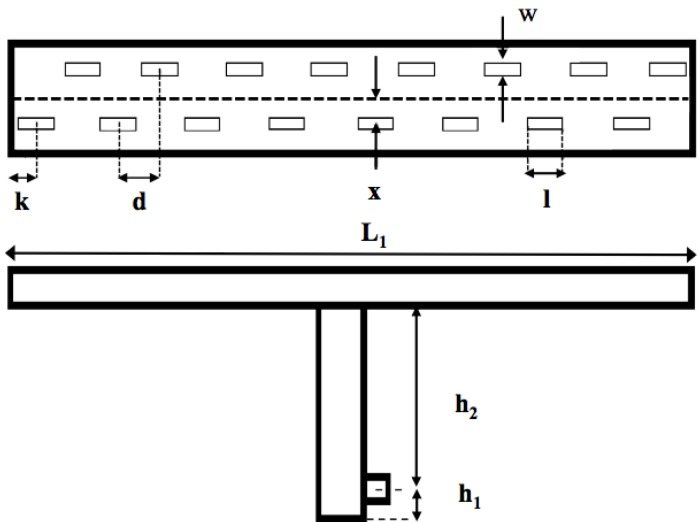
Main geometrical parameters of the transmitting antenna.

To create such a radiation pattern, a slot antenna consisting of a metallic T-shaped open waveguide with N small apertures suitably cut on the wider wall of the structure has been chosen, as shown in Figure 3. The N slots represent the radiating elements whose radiation patterns can constructively or destructively interfere with each other in order to obtain a single narrow radiation pattern according to the design requirements.

According to antenna theory [12,13,14], it is possible to estimate the number of slots and the distance between them, which are functional to achieve the desired aperture angle. In particular, considering a uniform linear array, the choice of N = 16 elements at half wavelength distance leads to the desired HPBW ≈ 4.7° but to a S_LL_ ≈ −13.7 dB that does not satisfy the design requirement. 

Therefore, in order to improve the antenna radiation performance, it has been necessary to suitably shape the E field radiated by each slot in order to obtain a triangular distribution of the field inside the structure. This is possible by exploiting the attenuation that affects the E field inside the waveguide due to the radiation from each slot. 

To reach this goal, a careful optimization of the main geometrical parameters of the array has been carried out using commercial EM software [15]. The parameters taken into account are defined in Figure 3 and their optimized values are summarized in Table 1.

**Figure 4:**
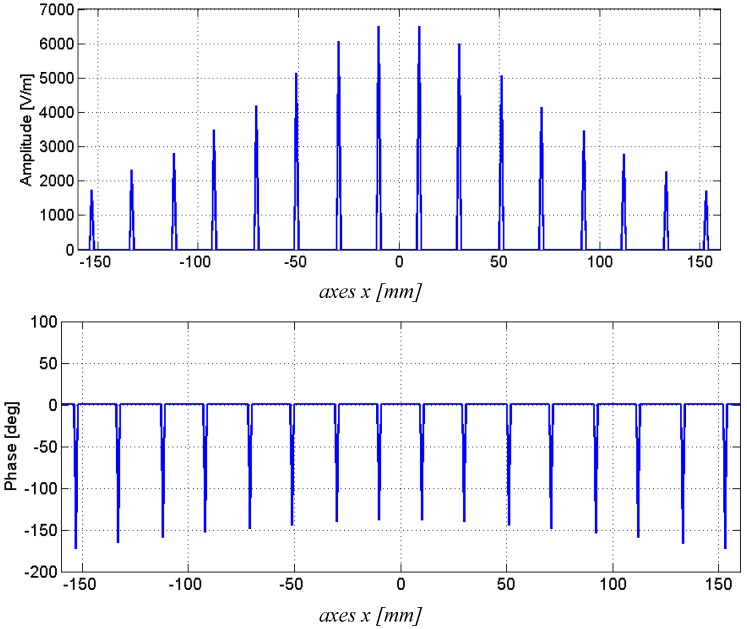
Amplitude (**Top**) and phase (**Bottom**) of the E field radiated by each slots obtained with the optimized parameters.

Moreover, Figure 4 clearly depicts the final E field distribution obtained inside the waveguide, highlighting a triangular configuration of the amplitude and a quite constant distribution of the phase (broadside radiation pattern). 

The optimized parameters have been obtained considering a work frequency range 10–11 GHz. This choice is a tradeoff between antenna radiating characteristics and reduced dimensions.

**Table 1:** Dimensions (mm) of Geometrical Parameters of the Transmitting Antenna.

Waveguide Length *L_1_*	328.1
T-junction length *(h_1_ + h_2_)*	93.8
Slot length *l*	17.0
Slot width *w*	1.0
Distance between the centers of two adjacent slots *d*	20.5
Distance between the center of a slot and the horizontal axes *x*	7.0
Distance between the last slot and the end of the structure *k*	10.2

It is worth noting that special attention has been paid to the design of the T-junction, which works as a power splitter, because it could lead to mismatching issues.

**Figure 5:**
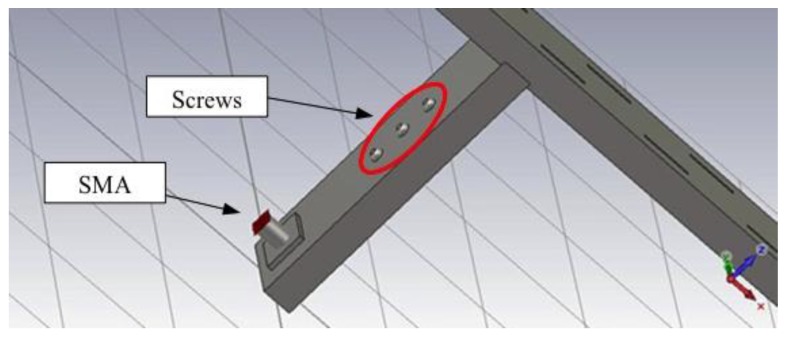
Screen shot of the antenna model showing the three screws.

Moreover, as shown in Figure 5, three screws have been added because different combinations of their depths of insertion could allow a fine-tuning of the resonance frequency and consequently an extremely accurate alignment between the resonance frequencies of the transmitting and receiving units, if required.

**Figure 6:**
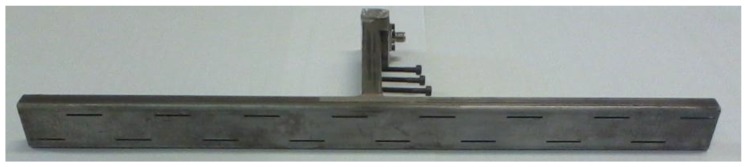
Picture of one of the transmitting slot antenna realized.

Finally, the optimized antenna has been realized and its main parameters (S_11_, radiation patterns and gain) have been measured and compared with the numerical values obtained by the EM solver. Figure 6 shows a picture of the final structure.

**Figure 7:**
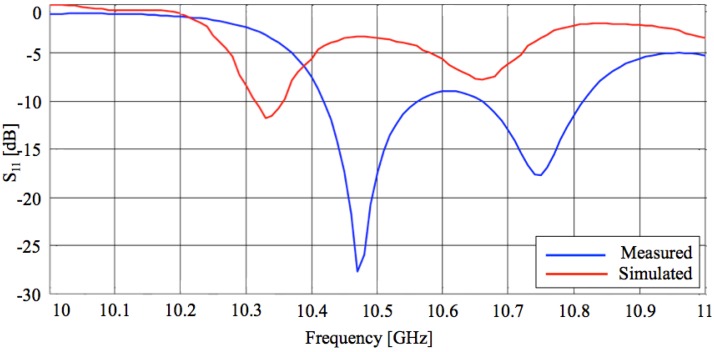
Reflection coefficient of the transmitting antenna: a comparison between calculated and measured values.

Figure 7 depicts a comparison between the simulated and measured values of the reflection coefficient, both refer to the final antenna model including the screws but without tuning. According to the working frequency of the power emitter, the attention has been focused on the peaks of resonance closer to 10 GHz. It is clear how although there is a slightly difference between measured and numerical reflection coefficients, the realized antenna performs a better impedance matching. It exhibits a bandwidth in the range 10.4–10.8 GHz, with a very good resonance (S_11_ about −30 dB) at about 10.47 GHz. The shift of about 150 MHz with respect to the simulated one (about 10.30 GHz) is probably due to mechanical tolerances and to the non-idealities of the material used to realize the antenna (not taken into account during the EM simulation). 

**Figure 8:**
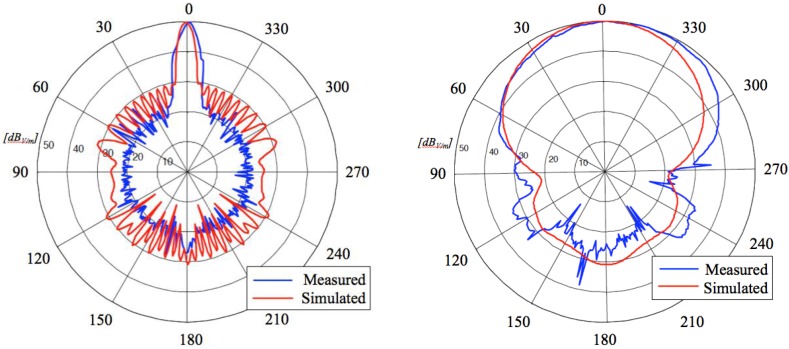
Radiation patterns of the transmitting slot antenna over both horizontal (**Left**) and vertical (**Right**) planes: a comparison between calculated and measured results.

Figure 8 shows the radiation patterns in dB over both H- and E-planes, calculated at the resonance frequency, and Table 2 summarizes the values of the HPBW (−3 dB) and the S_LL_ for both planes. 

The main evidence is the quite good matching between numerical and measured results and the satisfying agreement with design requirements (an HPBW of about 7° and a S_LL_ < −20 dB over H-plane). As concerns the radiation gain, the measured value is about 15 dB. That is a satisfactory value considering that no restrictive requirements exist for antenna gain.

**Table 2:** Radiation parameters of transmitting antennas: comparison between calculated and measured values.

	Angular Width (−3 dB) (Degree)	SLL (dB)
H-PLANE		
Numerical	5.6	−18.7
Measured	7.0	−23.0
E-PLANE		
Numerical	61.0	−19.1
Measured	76.0	−16.5

As mentioned in the introduction, for paths characterized by tight curves, a commercial horn antenna has to be preferred. In particular, during the test described in Section 3, the Flann Microwave Instrument 16240 horn has been used, whose main characteristics are: a good impedance matching from 8.20 GHz to 12 GHz with a mid-band gain of 20 dB and HPBW of about 35° over the E-plane and about 30° over the H-plane. 

#### 2.1.2. Receiving Unit

The receiving unit has the important role of communicating with the athlete. In fact, when the user is getting closer to one of the two EM walls, it has to alert the athlete so as he is driven to move toward the central position. As depicted in Figure 2, the receiving subsystem is composed of an antenna and a signal-processing unit. Since the running athlete should wear the receiving unit, the antenna and the circuit board for signal processing have to be small, compact, and lightweight. In the following, the components depicted in Figure 2 will be described in details.

##### Receiving Antenna

In order to satisfy the requirements of a lightweight and compact device, the antenna proposed is a small patch of four elements. The outline for microstrip antenna design assumes as known the dielectric constant of the substrate ε*_r_*, the resonant frequency *f_r_* and the height of the substrate *h* [16]. Therefore a commercially available dielectric substrate has been chosen and the same resonance frequency of the transmitting antennas has been taken into account: the ROGER 5870, with a thickness of 1.57 mm, a dielectric constant of 2.33 and loss tangent of 0.0012. A resonance frequency of *f_r_* = 10.47 GHz have been considered, accordingly with the working frequency of the transmitting antennas. Based on these data, the width *W* and the length *L* of the single element of the array have been computed:

(1) $$W=\frac{c}{2f_{r}}\sqrt{\frac{2}{\varepsilon_{r}+1}}$$

where *c* is the free-space velocity of light and ε*_r_* is the dielectric constant of the substrate, while

(2) $$L=\frac{1}{2f_{r}\sqrt{\varepsilon_{r\_eff}}\sqrt{\mu_{0}\varepsilon_{0}}}-2\Delta L$$

where ε*_r_eff_* and ∆*L* are introduced to take into account to the fringing effect and the wave propagation in the line [16]. 

**Figure 9:**
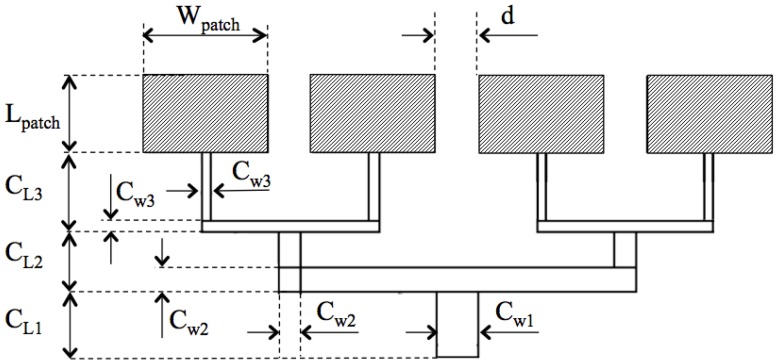
Schematic representation of the feeding network line.

Finally, in order to feed each single patch element, a corporate-feed network has been used together with a quarter wavelength configuration because it allows simply changing the network impedance, varying the width of the microstrip line. In particular, the impedance matching network has been designed so as to offer good matching at 50 Ω, that is the characteristic impedance of the SMA connector at the input of the microstrip antenna. To this end, the lengths of the feeding lines have been initially set to λ/4 (where λ is the wavelength relative to resonance frequency), then an optimization process has been carried out starting from the values obtained from theory (Equations (1) and (2)) and the final configuration is depicted in Figure 9. 

**Figure 10:**
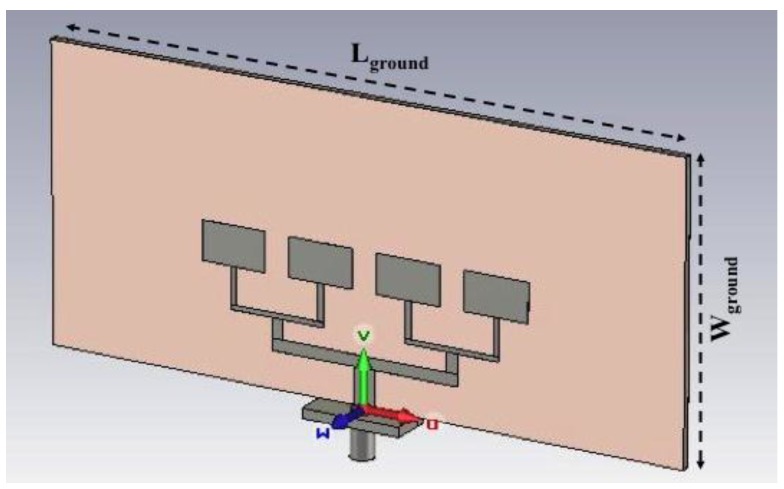
CAD model of the receiving patch antenna designed using commercial software [15].

Moreover, Figure 10 shows the antenna model designed using commercial EM software [15]. In Table 3 the main dimensions of the whole structure are summarized. 

**Table 3:** Dimensions (mm) of the geometrical parameters of the receiving antenna.

Length of ground plane L_ground_	130
Width of ground plane W_ground_	60
Length of patch L_patch_	13
Width of patch L_patch_	8.0
Length of 50 Ω C_L1_	7.5
Length of 100 Ω C_L2_	6.4
Length of 142 Ω C_L3_	8.5
Width of 50 Ω C_W1_	4.5
Width of 100 Ω C_W2_	2.4
Width of 142 Ω C_W3_	1.0

**Figure 11:**
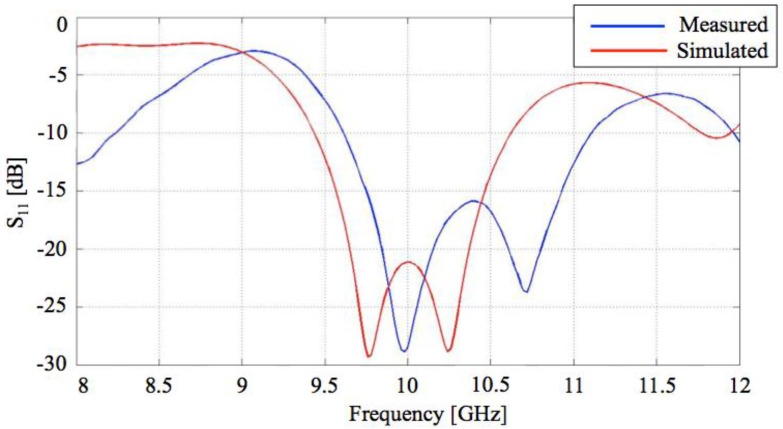
Reflection coefficient of receiving patch antenna: a comparison between calculated and measured values.

**Figure 12:**
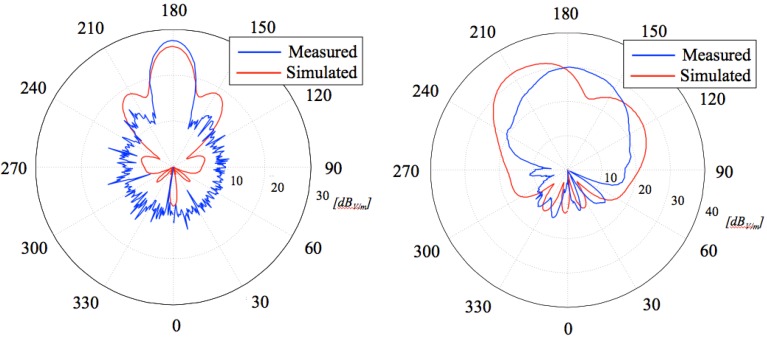
Radiation patterns of the receiving patch antenna over both horizontal (**Left**) and vertical (**Right**) planes: a comparison between calculated and measured results.

Once designed and optimized, the patch antenna has been realized in our laboratory. According to what has been done for the transmitting elements, the antenna reflection coefficient and the radiation patterns have been numerically calculated and experimentally measured and then compared, as shown in Figure 11 and Figure 12. The S_11_ trend is almost the same in both cases, although there is a shift of about 2% between the measured and simulated results probably due to the non-idealities of the material used to realize the patches as already observed for the transmitting antenna. 

Good impedance matching obtained for both transmitting and receiving antennas (see Figure 7 and Figure 11) and the wide frequency bandwidth of the latter (about 1.5 GHz), allowed the use of the T-shaped waveguide without the need for precision tuning of the resonance frequency. This means that *f_r_* = 10.47 GHz has been chosen as the working frequency.

The measured radiation patterns over both H- and E-planes are slightly different from the ones numerically calculated. In fact, as the figures clearly show and Table 4 summarizes, the performances of the realized antenna are even better in terms of directivity and side lobe level. Concerning the radiation gain, the measured value is about 11.8 dB that is a satisfactory value considering the antenna configuration and the type of application. 

**Table 4:** Radiation parameters of receiving antenna: Comparison between calculated and measured values.

	Angular Width (−3 dB) [Degree]	SLL[dB]
H-PLANE		
Numerical	19.4	−7.7
Measured	22.0	−14.0
E-PLANE		
Numerical	54.0	−18.7
Measured	51.0	−15.4

##### Signal Processing Unit

The antenna output is connected to a demodulator and an analog circuitry for the detection of the two-channel intensity levels. Due to the high operating carrier frequency, an active RMS power detector has been used as demodulator. The LTC5582 is an integrated circuit capable of converting the RF power level entering the input port into a voltage value proportional to the power expressed in dB. 

In general, the demodulator output will be the weighted sum of two square waves, at the two generated different modulation frequencies 1 kHz and 10 kHz whose relative amplitudes depend on the level of the signal received from the left and right transmitting antenna. This means that when the receiver faces one transmitting antenna more than the other, the intensity of the corresponding detected square wave is higher.

After a first stage of common amplification, the baseband signal splits into two active-band pass filters of the second order (Single Amplifier Biquadratic filters) to discriminate the fundamental component, at 1 kHz and 10 kHz respectively, and then both signals are buffered and rectified. The resulting voltage levels for the left and right side boundaries are converted every 20 ms into two 8-bit digital values and a first order IIR digital low-pass filter is used to avoid spikes and glitches.

**Figure 13:**
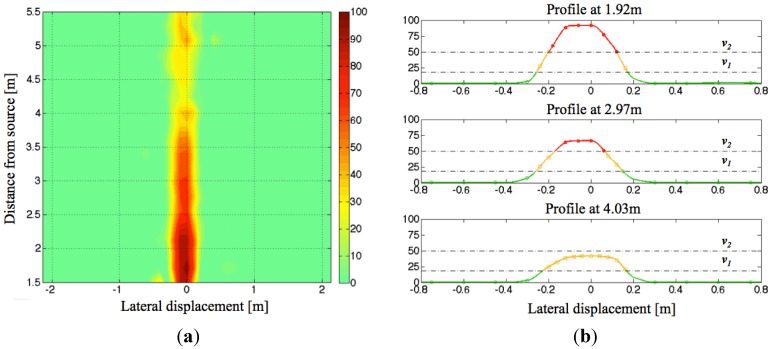
(**a**) Radiation map measured by the receiving subsystem on the 1 kHz channel, with antenna placed at 1m height from the floor. The color bar refers to the 8-bit analog to digital values numerically filtered after the amplification and selection chain and normalized to 100; (**b**) Profiles of the radiated field, measured at different distances from the transmitting unit. The dashed lines indicate the two different thresholds.

Figure 13 shows the experimental normalized voltage for the 1 kHz channel, after the ADC filtering, as a function of the receiving antenna position.

Then a calibration procedure has been implemented to define, at runtime, two thresholds for each channel. The two thresholds (dashed lines in the three profiles on the right of Figure 13) are computed as *v*_1_ = 18% × *v_ref_* and *v*_2_ = 50% × *v_ref_* and stored in the non-volatile memory of the microcontroller.

During normal operation, the receiving system output drives two vibration motors placed on both arms of the user. For each of them, three states of functioning are defined: no vibration if the measured filtered value is below *v*_1_; intermittent vibration if it is between *v*_1_ and *v*_2_; continuous vibration if it is above *v*_2_. A little hysteresis has been implemented to avoid bouncing between two adjacent states.

Finally, to prevent the user from getting too close or too far from the transmitting moving unit, a MaxBotix 7081 ultrasound sensor has been added to the system. This sensor warns the runner acoustically if he is approaching the vehicle (high pitch beeping sound) or if he is getting away from it (low pitch beep), while it is normally silent in the range from 1.5 m to 5 m. The whole receiving system is powered by a single rechargeable Li-Ion battery (3.7 V, 2000 mA·h) and power consumption is very low when only the receiver and MCU are active (90 mA), but obviously it increases when at least one motor is vibrating continuously (about 330 mA). Even assuming the highest current consumption, the expected duration of the battery before recharge is 6 h which is enough for a standard marathon race or training session.

As the EM safety issue is concerned, it is worth noting that at the given power and antenna gain, the exposure to E field at a distance of 1m from one of the transmitting antenna is 3.1 V/m, well below the ICNIRP thresholds, that are 137 V/m for occupational people and 61 V/m for the general public [17].

## 3. Results and Discussion

The EM guiding system performance and its reliability have been tested thanks to the collaboration of an Italian blind athlete, Andrea Cionna, who holds the world record for the fast marathon run by a totally blind man and won two bronze medals in blind long-distance running at the Paralympic Games.

### 3.1. Set up

**Figure 14:**
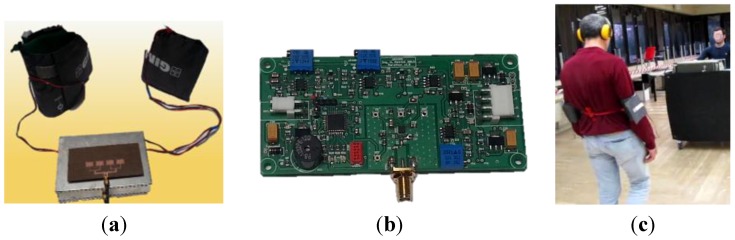
(**a**) The receiving unit worn by the user. It consists of a patch antenna, a metallic box containing the signal processing unit and two vibrational armbands; (**b**) Picture of signal processing board; (**c**) Picture of the system in use.

The system has been tested on increasingly difficult paths set up in an approximately 20 m × 17 m room of an engineering facility. The transmitting system has been placed on a mobile unit, which was manually pulled off and positioned at a safe distance (about 3 m) in front of the athlete who has been equipped with the receiving unit, as shown in Figure 14.

At first, a training time was required to instruct Cionna on how to figure out the vibro-tactile warnings coming from his right and left arms and the sound warning. Then a lot of tests with different paths were carried out, equipping the athlete with passive noise-reduction earmuffs in order to limit the orientation by hearing and to prevent him from following the noise arising from the wheels of the mobile unit.

### 3.2. Tests

**Figure 15:**
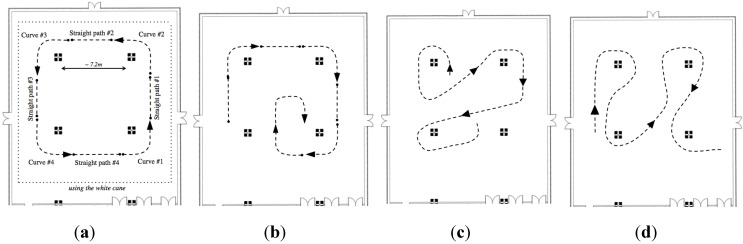
Schematic representations of different types of path performed in a big room (about 20 m × 17 m) room of an engineering facility. (**a**) Simple turn along room perimeter. (**b**) A turn along room perimeter and a final U-turn. (**c**,**d**) Complex paths with different curves and U-turns.

Some of the tests carried out with the collaboration of the blind volunteer are listed below and the relative movies can be watched on line [18]:

Test 1: Counterclockwise path type 1: simple turn along room perimeter Figure 15a (without earmuffs).
Test 2: Clockwise path type 1 Figure 15a (without earmuffs).
Test 3: Path type 2 a turn along room perimeter and a final U-turn, Figure 15b (without earmuffs).
Test 4: Counterclockwise path type 1 Figure 15a (with earmuffs).
Test 5: Path type 2 Figure 15b, Figure (with earmuffs).
Test 6: Path type 3: complex path with many curves and U-turns, Figure 15c (with earmuffs).
Test 7: Path type 4: complex path with many curves and U-turns, Figure 15d (with earmuffs).

The blind athlete performed each path exhibiting repeated changes of direction in the presence of curves, faithfully interpreting the vibro-tactile signals coming from his right and left arms. Path by path new curves have been added without informing the user, in order to disorient him and to make sure that he was performing the test exclusively following the device and not his memory.

Significant results can be obtained comparing the time required to complete each test and the relative mean velocity. Table 5 and Table 6 summarize these values for all the tests listed above. To better understand, the tests have been divided into two groups: simple paths and more complex paths. Table 5 concerns the first three tests whose lengths and complexity are comparable, while Table 6 concerns the more complex paths, whose comparison is more difficult because of the variability of straight paths, curves, and U-turns among tests.

First evidence is that, test by test, the athlete becomes more and more confident with the system; in fact, looking at tests 1, 2, and 3 performed without using the earmuffs, the time required for the complete path gradually reduces and the mean velocities increase.

Then, analyzing tests 1 and 4, which are the same path but performed respectively without and with the passive noise-reduction earmuffs, two evidences can be highlighted. On one hand, as expected, the time increases and the velocities decrease because the athlete cannot use his hearing anymore and consequently he is slightly more hesitant; on the other hand he is still able to walk quite fast and without bumps, meaning that he can satisfy achieving tasks exclusively using the EM guiding system. A similar comparison can be carried out between tests 3 and 5.

Moreover, comparing all the tests carried out with earmuffs, tests 4–7, it is evident that the mean velocity tends to increase, with the only exception of the last test, probably because of its extreme complexity and the exhaustion of the person who was operating the mobile unit. This means that the user, even completely isolated from the surroundings and after a very short training time to become confident with the new technology, is able to perform any unknown path.

Finally, to assess the potentialities of the EM guiding system, its performances have been compared with those of the user’s traditional white cane.

In particular, using the white cane the athlete needed to walk following a reference, repeatedly looking for the contact with the ribbon defining the perimeter of the room. The resulting path is depicted in Figure 15 as a pointed line: the curves became right angles, the movement insecure and hence the time required for a complete lap is extremely long (about 101 s) if compared with those required to perform the path of type 1 with or without earmuffs. On the other hand, using the EM system proposed, the user is able to gradually walk along the curves, with a good fluidity of movement.

**Table 5:** Comparison between paths of type 1 (simple paths).

Parameter	Test 1	Test 2	Test 4
Length (m)	52	52	52
Time (s)	60	58	65
Mean Velocity (m/s)	0.86	0.89	0.80
Mean Velocity (m/s) along straight paths	0.74	0.77	0.68
Mean Velocity (m/s) along curves	0.96	1.03	0.93

**Table 6:** Comparisons between more complex paths.

Parameter	Test 3	Test 5	Test 6	Test 7
Length (m)	51	61	71.50	65
Time (s)	50.46	71	67.29	75
Mean Velocity (m/s)	1.01	0.86	1.06	0.87

The results obtained, together with the user’s positive feedback, clearly demonstrates the effectiveness of the proposed system: the user’s pace is safe, constant, and fast as well as confirmed by the user himself, who declared, at the end of the trial, he always felt protected inside the EM walls.

## 4. Discussion

The research activity described in this paper has investigated the possibility of realizing a system able to let a visually impaired user to run autonomously. 

Therefore, the novelty of the paper lies on the smart use of well-known EM technologies and theories for a new field of application.

For a first step, laboratory instrumentation and homemade antennas have been used to set up the system and preliminary tests have been carried out with a blind end-user.

The encouraging results obtained demonstrate how the EM guiding system is able to actually let the blind volunteer walk autonomously and pave the way for the design of an optimized system.

In fact, bearing in mind user’s feedback, a series of improvements can be made: faster pace could be performed by installing the transmitting subsystem on a vehicle, as a car or a cycle, instead of pulling it manually; sound warnings coming from the ultrasound sensor could be replaced by variable vibrations to communicate to the blind athlete if he is too far or too close to the mobile unit, so as to avoid overcharging the sense of hearing; the size and weight of transmitting and receiving units could be reduced and their displacements optimized to improve comfort, fluidity, and agility.

## 5. Conclusions

The research activity presented in this paper wants to contribute to the reduction of limitations that visually impaired runners have to face during training or competitions.

To this end, EM technologies have been used to demonstrate the actual possibility of supporting blind runners without the presence of a sighted guide. The system has been set up mainly using laboratory instrumentation and homemade simple antennas, its capabilities have been demonstrated by tests carried out by a blind volunteer with encouraging results. 

It is our belief that such a system can pave the way for the realization of optimized and smart devices, based on EM technology, to support the autonomous mobility of people affected by visual diseases. For example, navigation in indoor environments represents a highly challenging task for the severely visually impaired that stimulates the interest of many research activities [19,20]. In this context, interesting opportunities may arise in joining EM technology and assistive robotics: autonomous mobile robots could be adapted to work as assistive robots, realizing an automated system able to guide a visually impaired subject along desired paths in complex indoor environments such as airports, stations, or hospitals. 
